# *Cladophora wrightiana* Var. *Minor* Extract Acts as an Adjuvant to Promote Natural Killer Cell Activation by Nasal Influenza Vaccine

**DOI:** 10.1002/fsn3.70807

**Published:** 2025-08-19

**Authors:** Thi Len Ho, So Yeon Ahn, Eun‐Ju Ko

**Affiliations:** ^1^ Interdisciplinary Graduate Program in Advanced Convergence Technology & Science Jeju National University Jeju Republic of Korea; ^2^ Department of Veterinary Medicine, College of Veterinary Medicine Jeju National University Jeju Republic of Korea; ^3^ Veterinary Medical Research Institute Jeju National University Jeju Republic of Korea

**Keywords:** *Cladophora wrightiana* var. minor (CW), dendritic cells (DCs), influenza virus, natural killer cells (NK), vaccine adjuvant

## Abstract

Natural killer (NK) cells, which are key components of the innate immune response, are crucial for ensuring the efficacy of vaccines as they rapidly eliminate infected cells and enhance the adaptive immune response, ensuring robust and lasting protection. In this report, we investigated the effect of *Cladophora wrightiana* var. *minor* (CW) extract, a marine alga, in activating NK cells, as an adjuvant to inactivated A/Puerto Rico/8/34 H1N1 influenza vaccine (iPR8). In vitro, CW extract significantly enhanced the level of activation markers CD69 and CD107a on NK cells and triggered intracellular secretion of interferon gamma (IFN‐γ) and granzyme B (GrB), indicating effective NK cell stimulation and cytotoxic function. In vivo, CW extract promoted substantial NK cell recruitment and activation, resulting in higher NK cell populations and elevated post‐immunization levels of activation markers. Additionally, CW extract increased IFN‐γ and GrB production in CD8^+^ T cells, highlighting its broader impact on the immune response. We also found direct evidence that CW‐activated NK cells and dendritic cells (DCs) interacted with and induced the activation of immature DCs and resting NK cells, respectively. These findings suggest that CW extract is a promising adjuvant for nasal vaccines, enhancing cellular immunity by activating NK cells and supporting interactions with DCs and CD8^+^ T cells.

## Graphical abstract

*Cladophora wrightiana var. minor* (CW) extract, administered as an adjuvant with inactivated influenza virus (iPR8), stimulates both innate and adaptive immune responses. CW enhances NK cell activation, cytotoxic function, and reciprocal crosstalk with dendritic cells, while also promoting CD8+ T cell responses and antigen‐specific IgG production. These findings support CW as a potent nasal vaccine adjuvant capable of boosting protective immunity. https://app.biorender.com/illustrations/6893f7d09e3fc89e9d953f76?slideId=2a6a42b6‐a3d9‐400a‐b839‐fa297b3108c5.

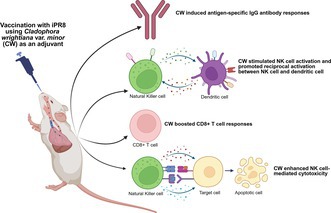

## 1 Introduction

Vaccination remains the most effective strategy for preventing viral infections, including influenza (Hussain 2019). Traditional influenza vaccines, built on whole‐virus inactivation, viral splitting techniques, or protein subunit platforms, are used to induce immunity (Wong and Webby 2013). However, the efficacy of these vaccines is limited by their inability to elicit a strong and sustained immune response on their own (Domínguez et al. 2016; Pollard and Bijker 2021); this necessitates the use of adjuvants (Fan et al. 2022; Zhao et al. 2023). Adjuvants enhance the immunogenicity of vaccines, leading to improved protection against the targeted virus by stimulating a more robust and longer‐lasting immune response (Marciani 2003; Pulendran et al. 2021).

Adjuvants primarily interact with the innate immune system, which provides immediate protection against pathogens (Awate et al. 2013; Coffman et al. 2010; O'Hagan and Valiante 2003). NK cells are key players in the innate immune response, as they recognize and destroy infected or transformed cells (Lam and Lanier 2017). Besides directly killing infected or abnormal cells, NK cells facilitate the link between innate and adaptive immunity via interactions with DCs (Cox et al. 2021; Rydyznski and Waggoner 2015). By activating NK cells, adjuvants enhance the overall effectiveness of influenza vaccines, promoting a more comprehensive immune response that includes both immediate and long‐term protection.

Traditional adjuvants such as aluminum salts (alum) have been used for decades to boost the humoral immune response to vaccines for diseases, such as tetanus, hepatitis B, and diphtheria (HogenEsch et al. 2018; MacLeod et al. 2011; Mbow et al. 2010; Tregoning et al. 2018). However, alum has limitations, including poor stimulation of cellular immunity and occasional local reactogenicity, such as pain and inflammation at the injection site (Singh et al. 2006). Another commonly used adjuvant, AS04, combines monophosphoryl lipid A (MPL) with aluminum salts, and is used in vaccines like Cervarix for HPV (Goetz et al. 2024). While AS04 enhances both humoral and cellular responses, MPL can cause local and systemic reactions, including pain, redness, swelling, fever, and malaise (Baldridge et al. 2000; Cluff 2010; Petrovsky 2015). MF59, an oil‐in‐water emulsion adjuvant used in influenza vaccines, enhances the immune response by recruiting immune cells and promoting antigen uptake by DCs, but it can have mild to moderate side effects, such as injection site pain, muscle aches, and headaches (Hervé et al. 2019; O Murchu et al. 2023).

Recently, adjuvants sourced from nature, including plant extracts, bacterial components, and marine algae, have shown significant promise in enhancing vaccine efficacy due to their low cost, favorable safety profiles, and immunostimulatory potential (Kumar et al. 2022; Lin et al. 2020; Pifferi et al. 2021; Spolaore et al. 2006). Compared to synthetic and traditional adjuvants, these natural compounds offer key advantages such as lower toxicity, biodegradability, and the ability to activate both innate and adaptive immune responses (Manilal et al. 2010; Spolaore et al. 2006; Woods et al. 2017). This makes them valuable in developing more effective and safer vaccines.

We have demonstrated that *Cladophora wrightiana* var. *minor* (CW), a green alga, exhibits immunostimulatory effects on DCs and macrophages (Ho et al. 2023). This research investigated the use of CW as an adjuvant to stimulate immune responses and increase the effectiveness of the inactivated influenza vaccine (iPR8). Specifically, we examined its ability to stimulate NK cell activation and promote interactions between NK cells, DCs, and CD8^+^ T cells following vaccination.

## 2 Materials and Methods

### 2.1 Mice, Vaccine, and Reagents

BALB/c female mice, aged 6–7 weeks, were purchased from SamTako (Korea). Animal experiments were conducted in accordance with protocols approved by the Institutional Animal Care and Use Committee of Jeju National University, Jeju, Korea (protocol number: 2021–0051).

Monophosphoryl lipid A (MPL; InvivoGen, USA) was used as a positive control in all experiments. The CW extract was provided by the Marine Bio Bank of the Marine Biodiversity Institute of Korea.

To prepare the inactivated A/Puerto Rico/8/34 H1N1 vaccine (iPR8), the PR8 virus strain (ATCC CCL‐34) was cultured in MDCK cells (Madin‐Darby canine kidney, NBL‐2, ATCC, USA). MDCK were maintained in DMEM (Gibco, Thermo Fisher Scientific, USA) containing 10% FBS (Corning) and 1× Antibiotic‐Antimycotic (Gibco, Thermo Fisher Scientific). Confluent MDCK monolayers were washed with 1× PBS and infected with PR8 virus at a multiplicity of infection (MOI) of 0.01 in serum‐free DMEM supplemented with 1 μg/mL TPCK‐treated trypsin (Sigma‐Aldrich Inc). The cells were then incubated at 37°C in 5% CO₂ for 1 h. Following viral adsorption, the inoculum was replaced with fresh medium, and the cells were further incubated for 3–4 days until cytopathic effects (CPE) became evident. The virus‐containing supernatant was clarified by centrifugation at 1500 rpm for 20 min and then inactivated with 1% formalin at 4°C for 1 day. The inactivated virus was concentrated by ultracentrifugation at 30,000 rpm for 1 h and resuspended in 1× PBS. The protein concentration was determined using a BCA protein assay kit (DoGenBio, Seoul, Korea), and the final product was stored at −80°C for use.

### 2.2 Mice Immunization

Mice were intranasally immunized with the iPR8 vaccine (2 μg) either alone or in combination with MPL (1 μg), CW (100 μg), or CW (200 μg) in a total volume of 50 μL PBS. Each experimental group consisted of four mice (*n* = 4). Briefly, mice were anesthetized using isoflurane administered via an oxygen‐controlled anesthesia system and received two intranasal doses of the vaccine formulation, with a 2‐week interval between the primary and booster immunizations. Mice were sacrificed 1 day after each immunization (prime and boost) to collect lung and spleen for analysis.

### 2.3 Cell Isolation From Lung and Spleen

Lung and spleen tissues from both naïve and immunized mice were collected and homogenized in RPMI 1640 medium (Roswell Park Memorial Institute 1640; Corning, NY, USA) using 70 μm cell strainers. The resulting homogenates were centrifuged at 1500 rpm for 5 min at 4°C, and the supernatants were discarded. Red blood cells were lysed from the cell pellets using ACK Lysing Buffer (Thermo Fisher Scientific). The cell suspensions were used for flow cytometry (FACS) and other experimental assays.

### 2.4 Preparation of NK Cells and DCs

NK cells were isolated from the spleen of mice using the EasySep Mouse NK Cell Isolation Kit (STEMCELL Technologies, Canada). To induce NK cell activation, the isolated cells were plated at a density of 5 × 10^5^ cells/mL in 6‐well plates and cultured for 48 h in RPMI 1640 medium with GlutaMax, supplemented with 10% fetal bovine serum (FBS), antibiotics, sodium pyruvate, non‐essential amino acids, and 2‐mercaptoethanol (10% LCM). Cells were treated with either 0.1 μg/mL MPL or 20 μg/mL CW to generate activated NK (ANK) cells. ANK cells were harvested, washed, and resuspended in 10% LCM for the next experiments.

Bone marrow‐derived dendritic cells (BMDCs) were prepared following a previously described protocol (Ho et al. 2023). After red blood cell lysis, the cells were cultured in RPMI 1640 medium containing 20 ng/mL recombinant mouse GM‐CSF (eBioscience, Invitrogen) at 37°C with 5% CO₂. The culture medium was replaced every 2 days. On day 6, immature dendritic cells (iDCs) were collected, seeded at a density of 5 × 10^6^ cells/mL in 6‐well plates, and incubated with either 0.1 μg/mL MPL or 20 μg/mL CW for 48 h to induce maturation.

### 2.5 Cytotoxicity Assay

NK cells were activated in vitro with CW (20 μg/mL) or MPL (0.1 μg/mL) for 2 days before use in cytotoxicity assays. In vivo‐activated NK cells were isolated from spleens 1 day after boost immunization.

YAC‐1 cells (Korean Cell Line Bank, Korea) were labeled with 2 μM CFSE (used as target cells), co‐cultured with NK cells from immunized mice or in vitro‐activated NK (ANK) cells at an effector‐to‐target (E:T) ratio of 5:1. After 4 h, cells were stained with Live/Dead‐Amcyan (Thermo Fisher Scientific), and dead target cells (CFSE^+^ Live/Dead^+^) were analyzed by flow cytometry.

### 2.6 In Vitro Assay of NK Cell–DC Interaction

Immature DCs (2 × 10^5^ cells/mL) were co‐cultured with activated NK (ANK) cells (4 × 10^5^ cells/mL), and resting NK cells (2 × 10^5^ cells/mL) were co‐cultured with mature DCs (1 × 10^6^ cells/mL) in 96‐well U‐bottom plates for 2 days. Prior to co‐culture, both CW‐ or MPL‐treated NK cells and DCs were washed one time with PBS to remove any residual stimulants. Supernatants were used to assess cytokine production, while cells were harvested for flow cytometry staining.

Dendritic cell activation was evaluated by staining with surface marker antibodies listed in Table S1, followed by analysis using a Beckman CytoFLEX LX.

### 2.7 Elisa

Immune sera were collected 1 day after the boost vaccination, and antigen‐specific antibody levels were measured using ELISA. ELISA plates were coated with iPR8 (500 ng/well) and incubated overnight at 4°C. The plates were blocked with 3% bovine serum albumin in phosphate‐buffered saline (PBS) containing 0.05% Tween 20. Serially diluted (1:100×, 1:1000×, 1:10000×, and 1:100000×) immune sera were added to the plates, which were further incubated for 2 h and subsequently washed with PBS containing 0.05% Tween 20. The levels of IgG, IgG1, and IgG2a antibodies were detected using horseradish peroxidase‐labeled secondary antibodies. Tetramethylbenzidine was used as a substrate, and absorbance was measured at 450 nm using a BioTek Synergy LX Multi‐Mode Reader (USA).

Cytokine levels in cell supernatants were detected by ELISA kits for IFN‐γ, granzyme B, IL‐12p70, and IL‐18 (R&D Systems, USA), as well as for TNF‐α, IL‐12p40, and IL‐15 (Invitrogen, USA), according to the manufacturers' instructions.

### 2.8 Cell Staining and Flow Cytometry

All cell samples, including both in vitro and in vivo‐derived lung and spleen cells, were first incubated with Fc blocker (anti‐mouse CD16/32) antibody (Clone 93; BioLegend) to block non‐specific antibody binding. Surface marker staining was then performed to assess the activation of NK cells, DCs, and T cells, using the antibodies listed in Table S1.

For intracellular cytokine staining, CD8^+^ T cells were analyzed without ex vivo restimulation with iPR8 antigen or influenza‐derived peptides, reflecting baseline activation status. The lung cells were first incubated with BD GolgiStop for 4 h at 37°C in a 5% CO_2_ incubator. After incubation, cells were harvested, washed, and blocked again with Fc blocker prior to surface marker staining. Cells were subsequently fixed and permeabilized using the Fixation/Permeabilization Solution Kit (BD Biosciences), followed by intracellular staining for cytokines.

Flow cytometry data were acquired using a Beckman CytoFLEX LX at the Bio‐Health Materials Core Facility, Jeju National University and analyzed using the FlowJo Software (Tree Star Inc.).

### 2.9 Statistical Analysis

All data are presented as mean ± SD. Statistical analysis was conducted using one‐way ANOVA followed by the Tukey's multiple comparison test. Pearson's correlation coefficient was employed to analyze the relationships between groups. A *p*‐value < 0.05 was considered to indicate statistical significance. All analyses were performed using the GraphPad Prism software version 9.2.0 (GraphPad Software Inc.).

## 3 Results

### 3.1 CW Stimulated NK Activation in Vitro

**FIGURE 1:**
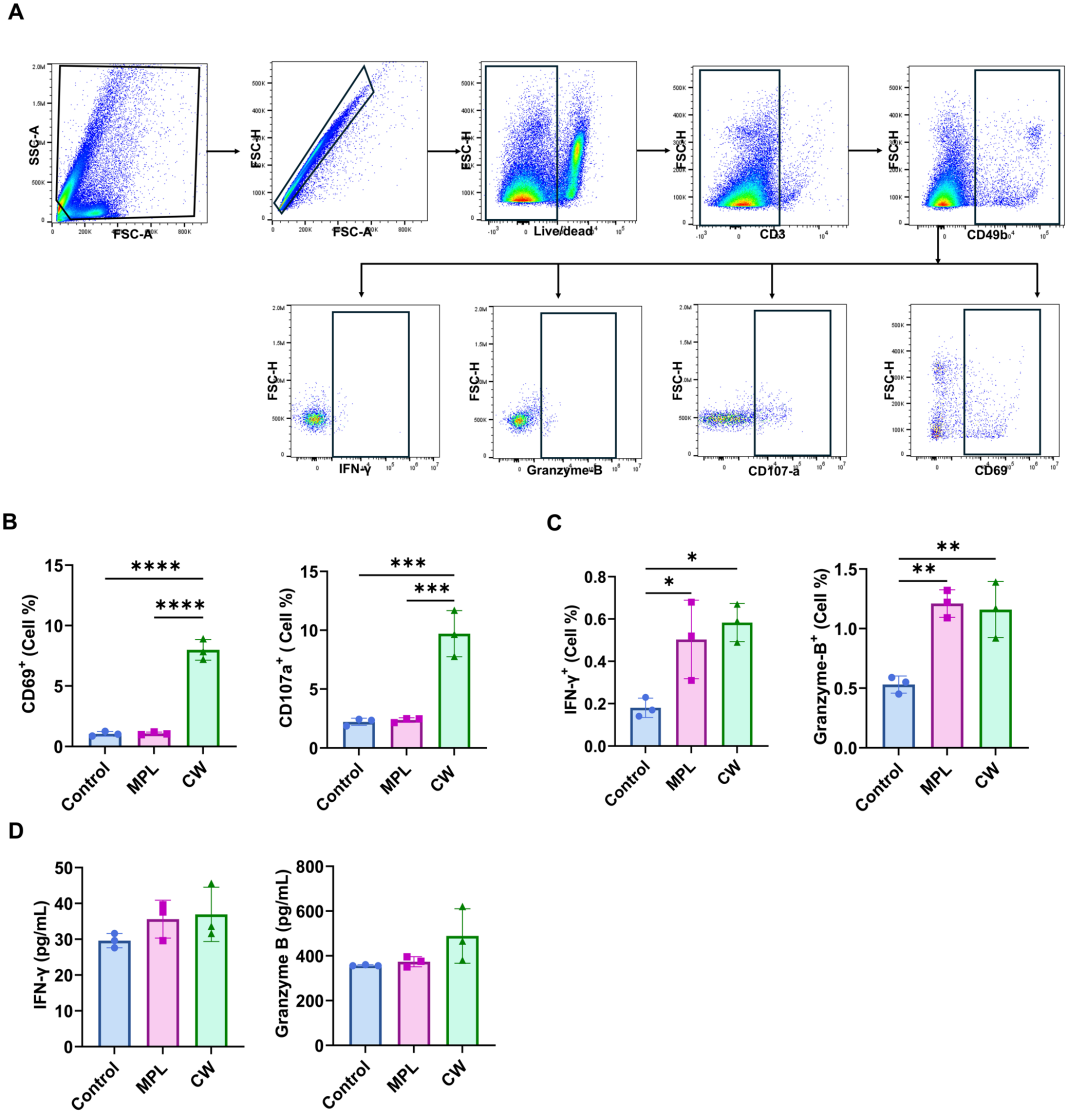
In vitro activation of NK cells by CW treatment. NK cells isolated from mouse splenocytes were treated with CW (20 μg/mL) or MPL (0.1 μg/mL). (A) The NK cell gating strategy is shown. (B) After 2 days of treatment, CD69 and CD107a expression on NK cells was assessed using flow cytometry. (C) Cytokine production was measured by intracellular staining, and (D) further quantified using ELISA. All data are expressed as mean ± SD. Statistical analysis between groups was performed using one‐way ANOVA and Tukey's multiple comparison test. *p < 0.0332, **p < 0.0021, ***p < 0.0002, and ****p < 0.0001.

CW extract had no cytotoxic effect on NK cells (Figure S1). CW extract significantly stimulated NK cell activation. CW treatment significantly increased CD69 and CD107a expression in NK cells compared to the control and MPL groups (Figure 1B). Additionally, NK cells stimulated with CW extract produced significantly higher amounts of intracellular IFN‐γ and GrB than those in the control or MPL‐treated groups (Figure 1C). While IFN‐γ and granzyme B levels were increased in the supernatant of CW‐treated cells, the differences were not statistically significant (Figure 1D).

### 3.2 CW Triggered Cytotoxicity Mediated by NK Cells in Vitro

**FIGURE 2:**
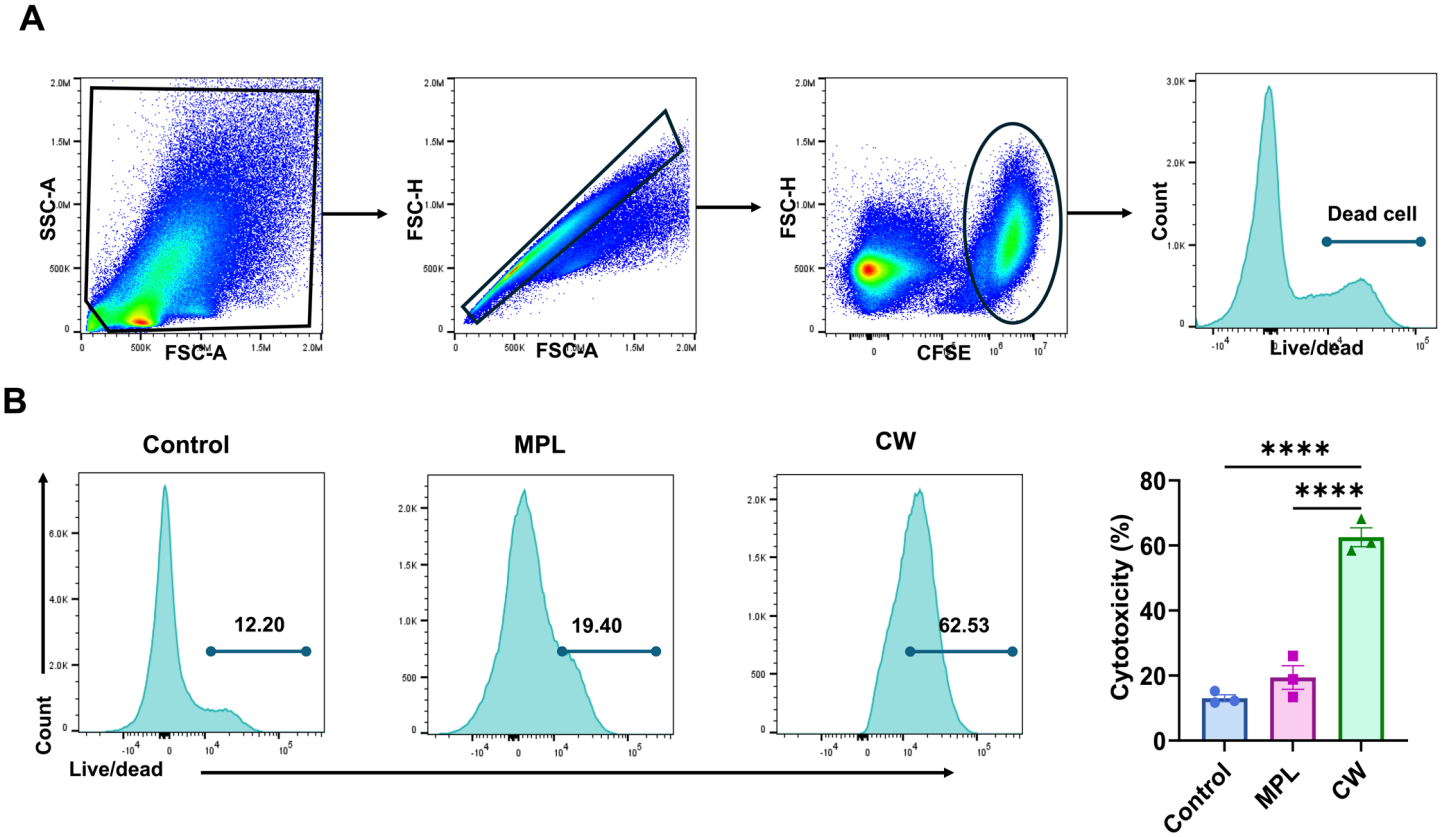
NK cell‐mediated cytotoxicity in vitro. NK cells isolated from mouse splenocytes were treated with CW (20 μg/mL) or MPL (0.1 μg/mL). After 2 days of treatment, the NK cells were co‐cultured with YAC‐1 target cells, and (A) cytotoxic activity was evaluated by quantifying the percentage of dead target cells via flow cytometry. (B) A representative histogram from the flow cytometry analysis is shown. All data are expressed as mean ± SD. Statistical analysis between groups was performed using one‐way ANOVA and Tukey's multiple comparison test. *p < 0.0332, **p < 0.0021, ***p < 0.0002, and ****p < 0.0001.

To assess the impact of CW extract on NK cell‐mediated cytotoxicity in vitro, NK cells were isolated from the spleens of naïve mice and stimulated with CW (20 μg/mL) or MPL (0.1 μg/mL) for 2 days. Following treatment, the NK cells were co‐cultured with CFSE‐labeled YAC‐1 target cells at a 5:1 effector‐to‐target ratio. The cells were stained with a Live/Dead dye, and the frequencies of dead target cells (CFSE^+^ Live/Dead^+^ YAC‐1^+^ cells) were determined by flow cytometry after a 4‐h incubation. NK cell‐mediated cytotoxicity in the CW‐treated group was significantly higher compared to the control and MPL‐treated groups (Figure 2B), indicating CW treatment enhanced cytotoxic activity.

### 3.3 CW Elicited Antigen‐Specific IgG Responses in Sera Following Vaccination

**FIGURE 3:**
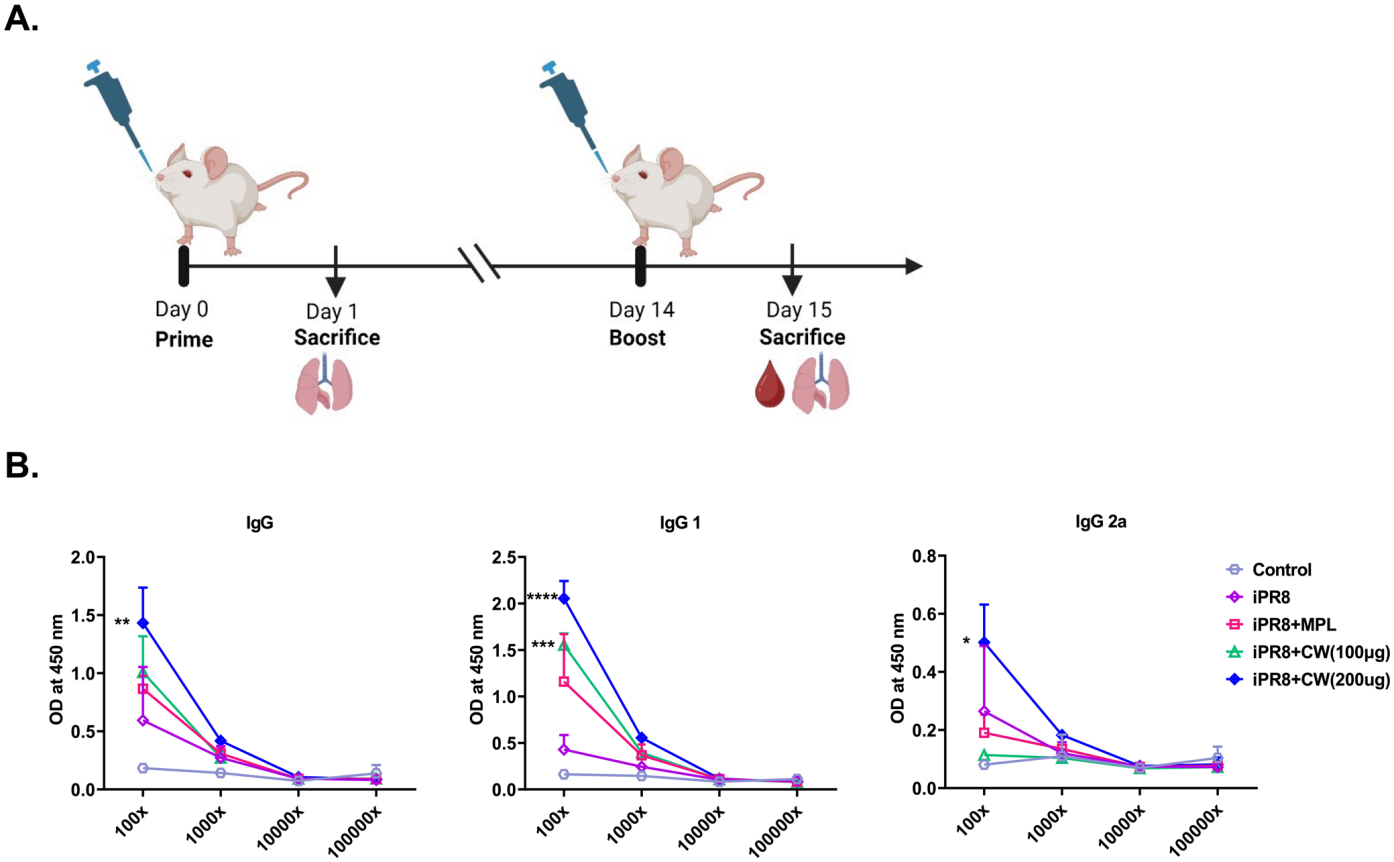
Production of antigen‐specific antibodies in serum after immunizations. (A) Mice were intranasally injected with the vaccine iPR8 (2 μg) either alone or combined with MPL (1 μg), CW (100 μg), or CW (200 μg), with immunizations administered twice at a 2‐week interval between the prime and boost doses. Sera samples were collected 1 day after the boost immunization. (B) These sera were then serially diluted, and the levels of antigen‐specific IgG, IgG1, and IgG2a antibodies were determined using ELISA. All data are expressed as mean ± SD. Statistical analysis between groups was performed using one‐way ANOVA and Tukey's multiple comparison test. *p < 0.0332; **p < 0.0021; ***p < 0.0002; and ****p < 0.0001 compared to the iPR8 group.

The schedule of immunization is presented in Figure 3A. Administration of iPR8 + CW (200 μg) elicited the strongest antigen‐specific IgG, IgG1, and IgG2a responses compared with other treatment groups, with significantly enhanced antibody levels at the 100× dilution compared with that in the iPR8 group (Figure 3B). The immune response was also enhanced in the iPR8 + MPL group compared to the iPR8 group; although not as effectively as in the CW group.

### 3.4 CW Promoted the Recruitment and Activation of NK Cells After Vaccination

Next, we collected lung samples from mice 1 day after both the prime and boost immunizations to assess how the CW extract affects NK cell recruitment and activation in response to vaccination.

**FIGURE 4:**
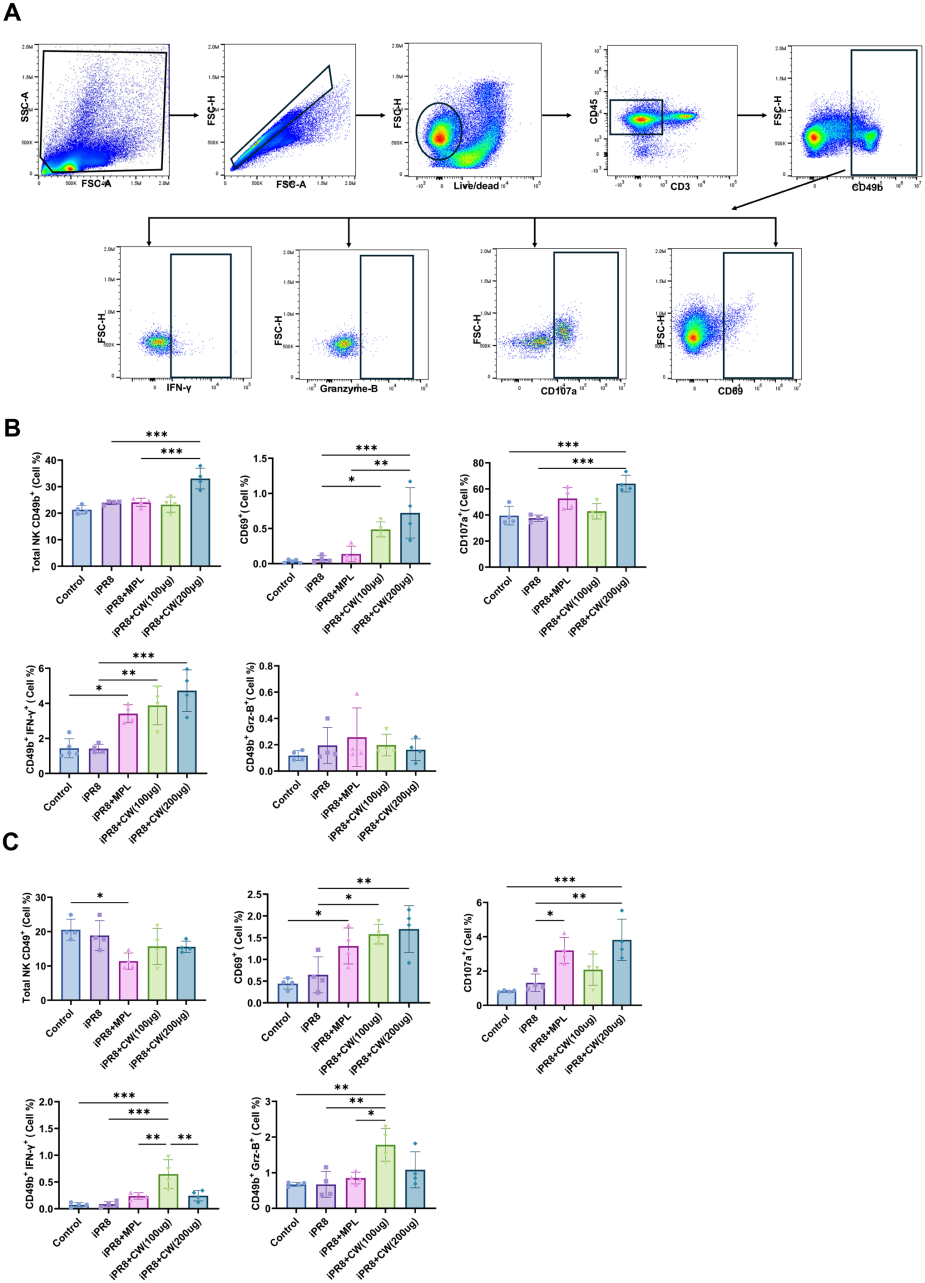
NK cell population after intranasal immunization with CW. Mice were injected with the vaccine iPR8 (2 μg) either alone or in combination with MPL (1 μg), CW (100 μg), or CW (200 μg) intranasally. The immunizations were administered twice, with a two‐week interval between the prime and boost doses. (B) On day 1 post‐prime and (C) boost immunization, the NK cell population in lung cells was analyzed using flow cytometry. Intracellular cytokine levels of IFN‐γ and granzyme B in NK cells of the lung were measured. (A) The NK cell gating strategy is shown. All data are expressed as mean ± SD. Statistical analysis between groups was performed using one‐way ANOVA and Tukey's multiple comparison test. *p < 0.0332; **p < 0.0021; ***p < 0.0002; and ****p < 0.0001.

1 day after the prime immunization (Figure 4B), the total NK cell population (CD45^+^CD3^−^CD49b^+^) and the expression of activation markers CD69 and CD107a were significantly increased in all CW‐supplemented groups compared to the control and iPR8 groups. Additionally, intracellular IFN‐γ levels in NK cells were significantly higher in the CW group than in the control and iPR8 alone group.

Following the boost immunization (Figure 4C), the total NK cell population remained significantly elevated in the CW‐treated group. The surface expression of CD69 and CD107a, as well as intracellular levels of IFN‐γ and granzyme B, was significantly elevated in the CW‐treated groups. Notably, the CW (200 μg) group exhibited higher expression of CD69 and CD107a, whereas the CW (100 μg) group induced greater production of IFN‐γ and GrB.

### 3.5 CW Enhanced Activation and Interaction of DCs and NK Cells After Immunization

**FIGURE 5:**
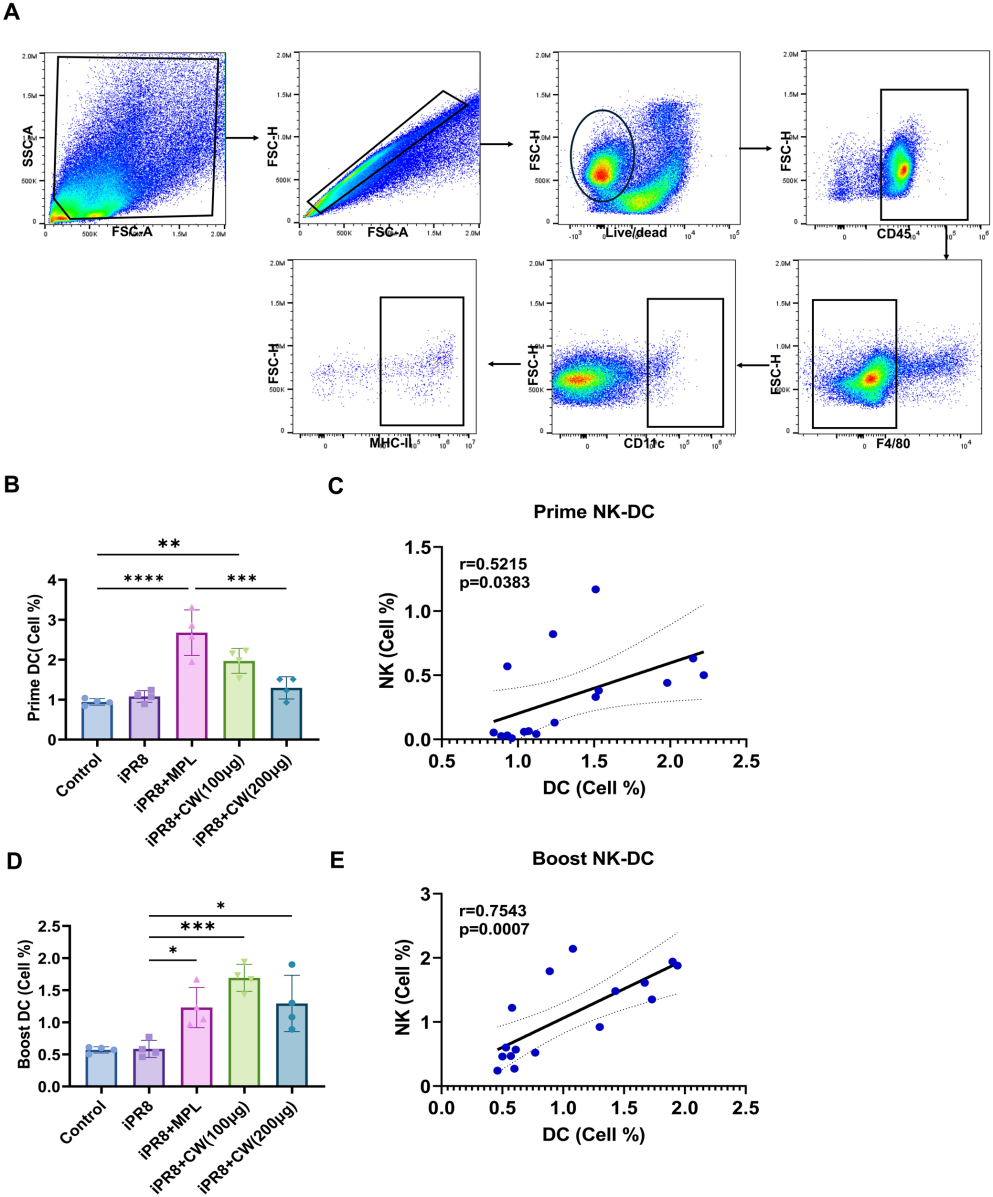
Correlation of DC and NK cell activation after intranasal iPR8 + CW immunization. Lungs were collected on day 1 after the prime (B, C) and boost (D, E) doses. DCs were identified as CD45^+^F4/80^−^CD11c^+^MHC‐II^high^ cells (A), and their frequencies were correlated with activated NK cells (CD49b^+^CD69^+^) (C, E). All data are expressed as mean ± SD. Statistical analysis between groups was performed using one‐way ANOVA and Tukey's multiple comparison test. *p < 0.0332, **p < 0.0021, ***p < 0.0002, and ****p < 0.0001. For correlation analysis, Pearson's correlation coefficient was used to analyze the correlations between groups. *p* < 0.05 was considered statistically significant.

We further examined if NK cell activation stimulates other cells, such as DCs, to enhance the immune response. Lungs were collected 1 day after both the prime and boost immunizations; cell phenotypes were analyzed by flow cytometry. The percentage of DCs (CD45^+^F4/80^−^CD11c^+^MHC‐II^high^) in the group vaccinated with CW (100 μg) and CW (200 μg) after prime (Figure 5B) and boost (Figure 5D) vaccination was significantly increased. Furthermore, a positive correlation was observed between DCs and NK cell (CD49b^+^CD69^+^) activation after prime (Figure 5C) and boost (Figure 5E) immunizations.

### 3.6 CW Increased Cytotoxic CD8 ^+^ T Cell Levels in the Lungs After Boost Vaccination, Showing a Correlation With NK Cell Frequency

**FIGURE 6:**
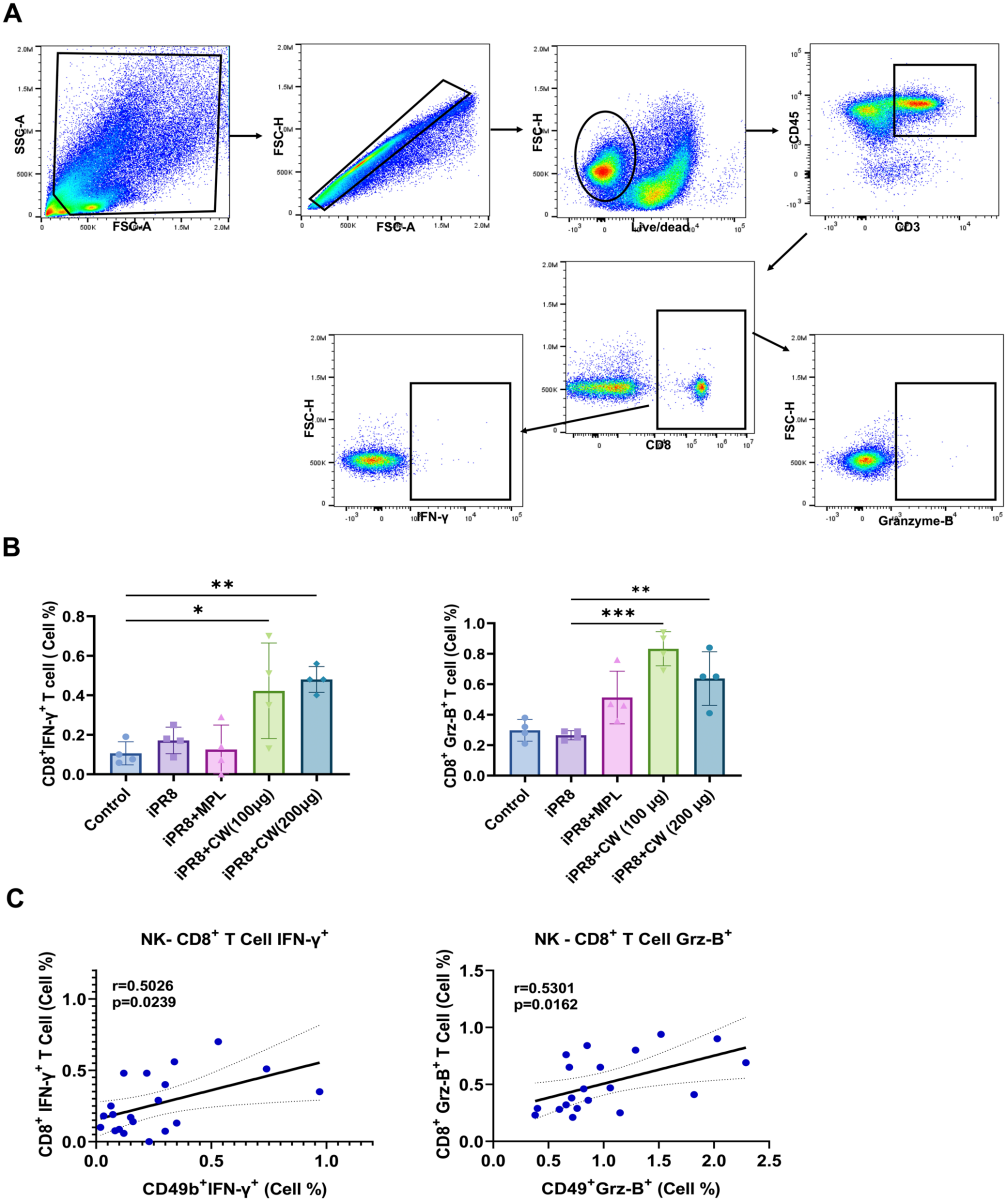
Cytotoxic CD8^+^ T cell expression of lung cells after boost vaccination and correlation between the percentage of CD8^+^ T cells and NK cells. Lungs were harvested at 1 day post‐boost vaccination, and the level of IFN‐γ and GzB in CD8^+^ T cells was gated and measured by flow cytometry (A, B). The correlation analysis between the population of CD8^+^ T cells and NK cells (C). CD8^+^ T cells were not restimulated ex vivo with iPR8 antigen or peptides prior to staining. All data are expressed as mean ± SD. Statistical analysis between groups was performed using one‐way ANOVA and Tukey's multiple comparison test. *p < 0.0332, **p < 0.0021, ***p < 0.0002, and ****p < 0.0001. Pearson's correlation coefficient was used to analyze the correlations between groups. *p* < 0.05 was considered statistically significant.

We next investigated if the activation of NK cells enhanced the adaptive immune response by investigating the activation of T cells, specifically CD8^+^ T cells, 1 day after boost immunization. Interestingly, the level of IFN‐γ and granzyme B in CD8^+^ T cells in the groups vaccinated with CW extract (100 and 200 μg) was significantly increased (Figure 6B). NK cell frequency showed a positive correlation with the activation of CD8^+^ IFN‐γ^+^ and CD8^+^ GrB^+^ T cells (Figure 6C).

### 3.7 CW Treatment Enhanced Reciprocal NK Cells Activation and DCs in Vitro

**FIGURE 7:**
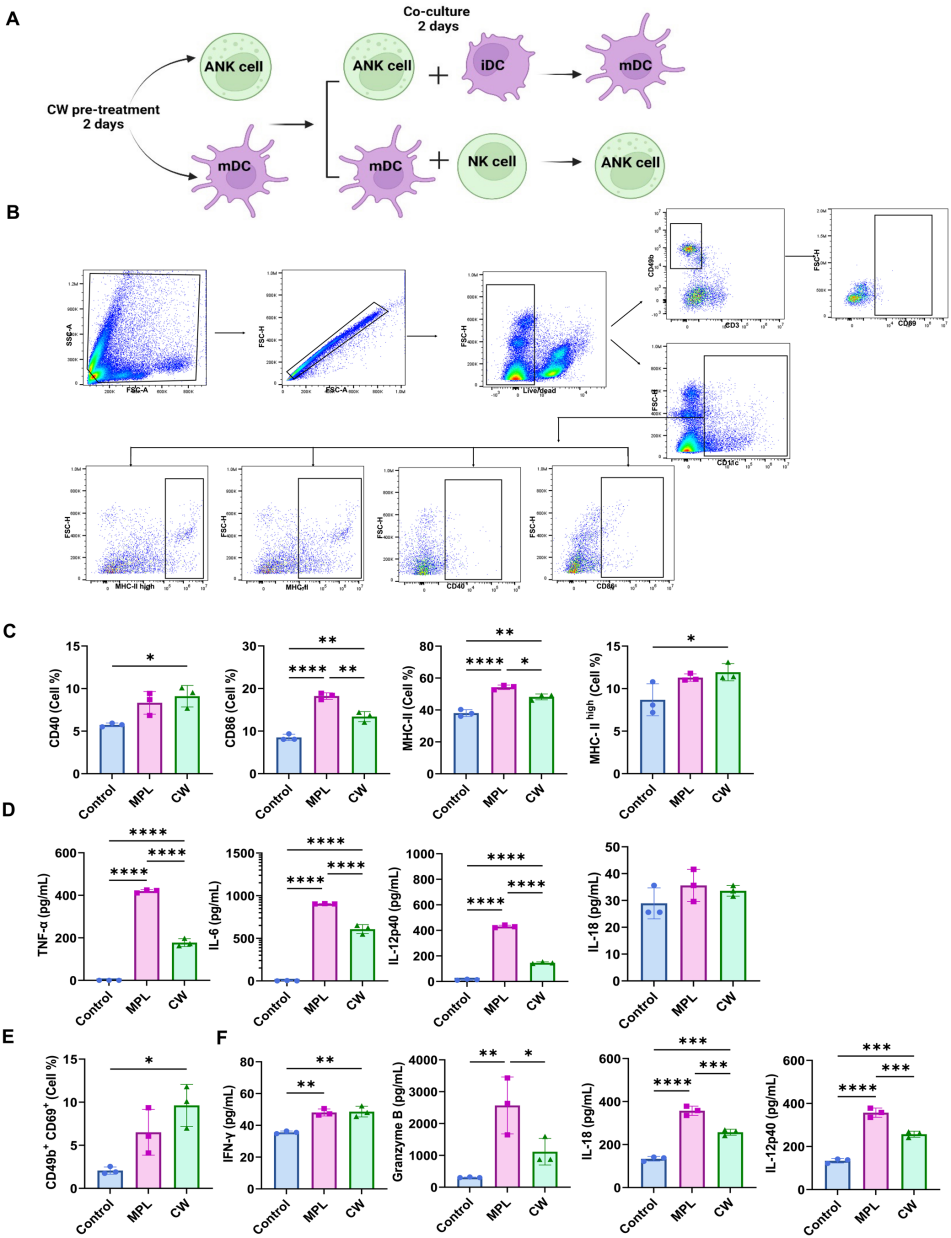
In vitro activation of DCs by CW‐activated NK cells and of NK cells by CW‐treated DCs. Immature DCs were co‐cultured with NK cells pre‐treated with MPL (0.1 μg/mL) or CW (20 μg/mL), while pre‐activated DCs with MPL (0.1 μg/mL) or CW (20 μg/mL) were co‐cultured with resting NK cells. After 2 days, the cells were harvested and analyzed for activation markers (CD86 and MHC‐II for DCs (C); CD69 for NK cells (E)) using flow cytometry. Cytokine production of DCs (D), NK cells (F) in the culture supernatants was quantified using ELISA. Gating strategy for DC and NK activation is shown (B). All data are expressed as mean ± SD. Gating strategy for DC and NK activation is shown (B). Statistical analysis between groups was performed using one‐way ANOVA and Tukey's multiple comparison test. *p < 0.0332, **p < 0.0021, ***p < 0.0002, and ****p < 0.0001.

To further investigate the crosstalk between NK cells and DCs, we conducted in vitro co‐culture experiments focusing on the CW‐treated group and comparing with the MPL group. Co‐culture of CW‐activated NK cells with iDCs (Figure 7C,D) and of CW‐activated DCs with resting NK cells (Figure 7E,F) increased the activation of both cell types. In the CW group, significant increases in DC maturation markers (CD86 and MHC‐II) and cytokine production (TNF‐α, IL‐6, and IL‐12p40) were observed when DCs were co‐cultured with CW‐activated NK cells (Figure 7C,D). Additionally, when NK cells were co‐cultured with DCs activated by CW extract, CD69 expression on NK cells was significantly increased, and secretion of IFN‐γ, GrB, IL‐18, and IL‐12p40 was enhanced (Figure 7E,F). In comparison, the MPL group also exhibited increased activation but to a lesser extent than the CW group.

## 4 Discussion

NK cells are one of the key effectors of innate immunity, capable of recognizing and destroying infected or transformed cells without prior sensitization, and are important in the context of vaccination. NK cells bridge innate and adaptive immunity by interacting with DCs and T cells (Raulet 2004; Sun and Lanier 2009). Activation of NK cells enhances vaccine efficacy by promoting a robust and comprehensive immune response (Wolf et al. 2023). In this study, we investigated CW as an adjuvant for influenza vaccination, focusing on its ability to enhance NK cell activation, recruitment, and interaction with DCs and CD8^+^ T cells, as well as its role in increasing antibody levels following vaccination. CW extract significantly induced antigen‐specific IgG antibody production, stimulated NK cell activation, enhanced NK cell‐mediated cytotoxicity, promoted DC maturation, and boosted CD8^+^ T cell responses.

Antibody production is a well‐established and essential component of the immune response to vaccination, serving as a critical mechanism for direct neutralization of viruses during infection (Kulkarni 2020; Pantaleo et al. 2022). In our study, iPR8 combined with 200 μg CW significantly enhanced IgG, IgG1, and IgG2a levels (Figure 3B). Although NK cell cytokine production (IFN‐γ, granzyme B) was higher in the 100 μg group post‐boost, the 200 μg group showed stronger overall antibody responses and higher NK activation markers (CD69, CD107a). Since serum was collected just 1 day after the booster, these responses likely reflect priming‐phase effects. These findings highlight a non‐linear dose–response, where lower doses favor NK cytokine activation and higher doses promote stronger adaptive responses. Although IFN‐γ is Th1‐associated, the similar increase in IgG1 and IgG2a suggests a mixed Th1/Th2 profile. As IL‐4 was not measured, we cannot rule out contributions from other IL‐4‐producing innate cells. Overall, CW appears to modulate immunity in a dose‐dependent, non‐linear manner. Future studies measuring IL‐4, antibody kinetics, IgG subclass ratios, and using NK cell depletion will help clarify how CW shapes coordinated innate and adaptive immune responses.

The activation of NK cells triggers the release of cytokines and cytotoxic substances, which are crucial for pathogen clearance and immune regulation (Abel et al. 2018; Paolini et al. 2015). Enhancing NK cell activation through adjuvants can potentiate vaccine efficacy by stimulating both immediate and long‐term immune responses (Bonam et al. 2017; Pashine et al. 2005). Our in vitro experiments revealed that CW extract significantly increased the level of activation markers CD69 and CD107a in NK cells (Figure 1B). Additionally, CW‐treated NK cells showed higher levels of intracellular IFN‐γ and granzyme B compared with control and MPL‐treated groups (Figure 1C). CW extract effectively stimulated the activation of NK cells, enhancing their cytotoxic function (Figure 2) and cytokine production. These findings demonstrate that CW extract effectively stimulates NK cell activation and effector function, supporting its potential as an innate immune‐stimulating adjuvant.

In the context of influenza vaccination, to recognize and eliminate virus‐infected cells and facilitate a robust immune response, activated NK cells produce cytokines, express activation markers on their surface, and promote cytotoxic activity (Dou et al. 2015). Our in vivo experiments showed that CW extract promotes significant NK cell recruitment and activation (Figure 4). Total NK cell population and expression of activation markers CD69 and CD107a were increased in CW‐treated groups after prime immunization (Figure 4B). These markers remained elevated post‐boost immunization, alongside increased cytokine levels (IFN‐γ and granzyme B) (Figure 4C). However, the total NK cell population decreased after the boost. This can be explained by several factors: initially, NK cells expand in response to the vaccine and adjuvant; but post‐boost, the immune system undergoes homeostatic regulation to prevent overactivation, resulting in contraction of the NK cell population (Hildeman et al. 2007). Additionally, NK cells might transition to a more effector‐driven phase, leading to cellular exhaustion or apoptosis. Redistribution of NK cells to other tissues may also contribute to the observed decrease (Goodier and Riley 2021). These enhancements suggest that CW extract boosts NK cell function, crucial for an effective immune response, supporting the potential of CW as a strong vaccine adjuvant. Furthermore, CW extract significantly increased NK cell‐mediated cytotoxicity following in vivo immunizations. The cytotoxicity assay revealed higher percentages of dead target cells in CW‐adjuvanted groups, indicating enhanced NK cell function (Figure S2). This result is critical, as it demonstrates the ability of CW extract to not only activate NK cells but also enhance their cytotoxic activity, essential for eliminating infected cells. Moreover, NK cells exhibited enhanced effector functions after booster immunization (Figure 4C), suggesting possible trained immunity or memory‐like behavior (Cooper and Yokoyama 2010). Although NK cells are traditionally considered innate effectors (Moretta et al. 2008), emerging evidence shows they can respond more strongly upon re‐exposure (Sun et al. 2010). While not directly assessed here, future studies will investigate whether CW induces such trained responses to enhance vaccine efficacy.

The communication between NK cells and DCs is important for a robust immune response, facilitating mutual activation and enhancement of each other's functions. NK cells can induce DC maturation, enhancing their ability to present antigens and produce cytokines essential for adaptive immune responses (Moretta et al. 2008). Conversely, mDCs can boost the activation of NK cells, increasing their cytotoxic activity and cytokine production. This reciprocal relationship ensures a coordinated and amplified immune response, crucial for effective pathogen defense and vaccine efficacy (Thomas and Yang 2016). In vitro co‐culture of CW‐activated NK cells with iDCs, or of CW‐activated DCs with resting NK cells, increased their activation levels (Figure 7). This reciprocal mechanism, more pronounced with CW than MPL, leads to a stronger immune response. In vivo, CW extract significantly increased DC prevalence and NK cell activation post‐vaccination (Figure 5). Positive correlations were observed between DC and NK cell activation; however, we did not assess DC maturation markers such as CD86 or CD40 upregulation in vivo. Since DC maturation is essential for effective antigen presentation and T cell priming, this represents a limitation in our current mechanistic analysis. Future studies will incorporate in vivo assessment of DC activation and maturation to further elucidate how CW enhances the NK‐DC‐T cell axis and contributes to improved adaptive immune responses following mucosal vaccination. Moreover, DCs in the lung were identified using the gating strategy CD45^+^F4/80^−^CD11c^+^MHC‐II^high^. However, we acknowledge that this approach may not fully exclude alveolar macrophages or monocyte‐derived cells, as these populations can share high CD11c and MHC‐II expression and show variable F4/80 levels depending on activation status. In future studies, additional markers such as Siglec‐F, CD103, CD11b, or CD64 will be included to better distinguish DC subsets from overlapping myeloid populations.

NK cells also play a key role in shaping the adaptive immune response by interacting with various cell types, such as DCs. These interactions influence the activity of NK cells and modulate the overall immune response. The communication between NK cells and DCs is particularly significant, as it can substantially affect the responses of both NK and T cells (Raulet 2004). Interestingly, we discovered that combining vaccination with CW significantly increased the levels of IFN‐γ and granzyme B in the CD8^+^ T cell population (Figure 6B). Secretion of IFN‐γ and granzyme B by NK cells correlated positively with the production of these molecules by CD8^+^ T cells (Figure 6C). Moreover, a significant correlation was observed between the total NK and CD8^+^ T cells 1 day after the boost vaccination (Figure S3). These data demonstrate functional parallels between NK cells and CD8^+^ T cells. When NK cells encounter their cognate ligands on target cells, they mediate cytotoxicity through the directional release of granzyme B and secrete effector cytokines such as IFN‐γ in a manner almost identical to that of effector and CD8^+^ T cells (Lanier 2005, 2007). However, as peptide restimulation was not performed, it remains unclear whether the observed CD8^+^ T cell responses were vaccine‐specific or reflected general activation. Further studies using influenza‐specific peptide stimulation or MHC class I tetramers will be necessary to confirm whether CW enhances antigen‐specific CD8^+^ T cell responses. In summary, CW extract enhances NK cell function and interaction with DCs and CD8^+^ T cells, boosting the overall immune efficacy.

## 5 Conclusions

This study demonstrated that CW is a promising adjuvant candidate for influenza vaccination owing to its ability to stimulate NK cell activation both in vitro and in vivo, increase NK cell‐mediated cytotoxicity, promote DC maturation, boost CD8^+^ T cell responses, and enhance the antigen‐specific antibody level in vivo. CW‐treated groups exhibited higher levels of IFN‐γ and granzyme B in CD8^+^ T cells. CW resulted in significant upregulation of antibody production, indicating a robust humoral immune response. These findings highlight the potential of CW to improve influenza vaccine efficacy while ensuring cellular safety, making it a valuable candidate for vaccine adjuvant development.

## Author Contributions

**Thi Len Ho:** conceptualization (equal), data curation (equal), formal analysis (lead), investigation (lead), methodology (lead), validation (lead), visualization (lead), writing – original draft (lead), writing – review and editing (lead). **So Yeon Ahn:** formal analysis (equal), investigation (equal), methodology (equal), writing – review and editing (equal). **Eun‐Ju Ko:** conceptualization (lead), data curation (equal), formal analysis (equal), funding acquisition (lead), project administration (lead), supervision (lead), validation (equal), visualization (equal), writing – review and editing (lead).

## Conflicts of Interest

The authors declare no conflicts of interest.

## Supporting information


**Data S1.** Supporting Information.

## Data Availability

The data supporting the findings of this study can be obtained from the corresponding authors upon reasonable request.
