# Supplementary material for: Cladophora wrightiana Var. Minor Extract Acts as an Adjuvant to Promote Natural Killer Cell Activation by Nasal Influenza Vaccine
Source: Food Sci Nutr. 2025 Aug 19;13(8):e70807. doi: 10.1002/fsn3.70807 (PMC12364720; doi:10.1002/fsn3.70807)
Supplement: Supplementary file 1 — Data S1. Supporting Information. [file FSN3-13-e70807-s001.docx]

**
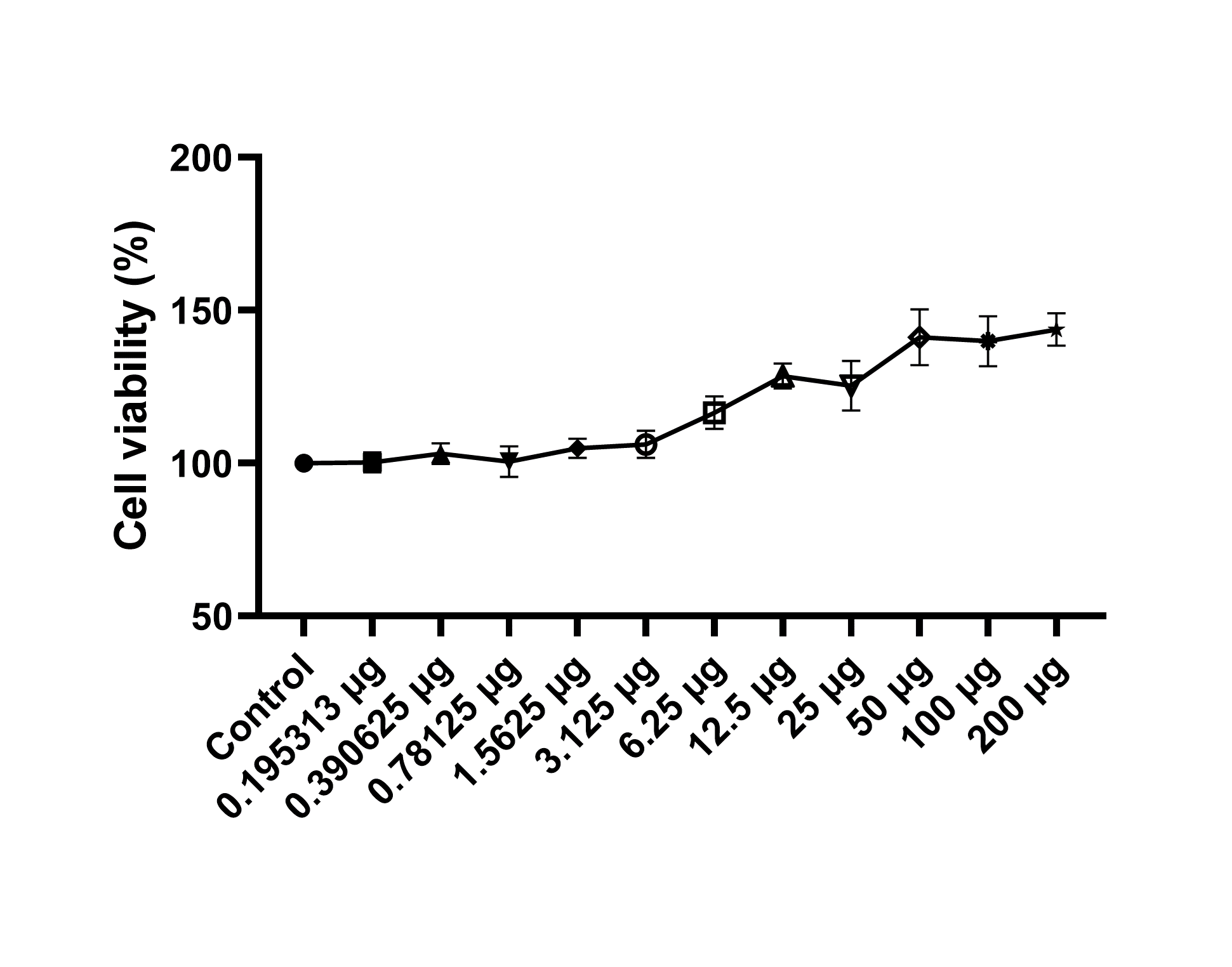
Supplements**


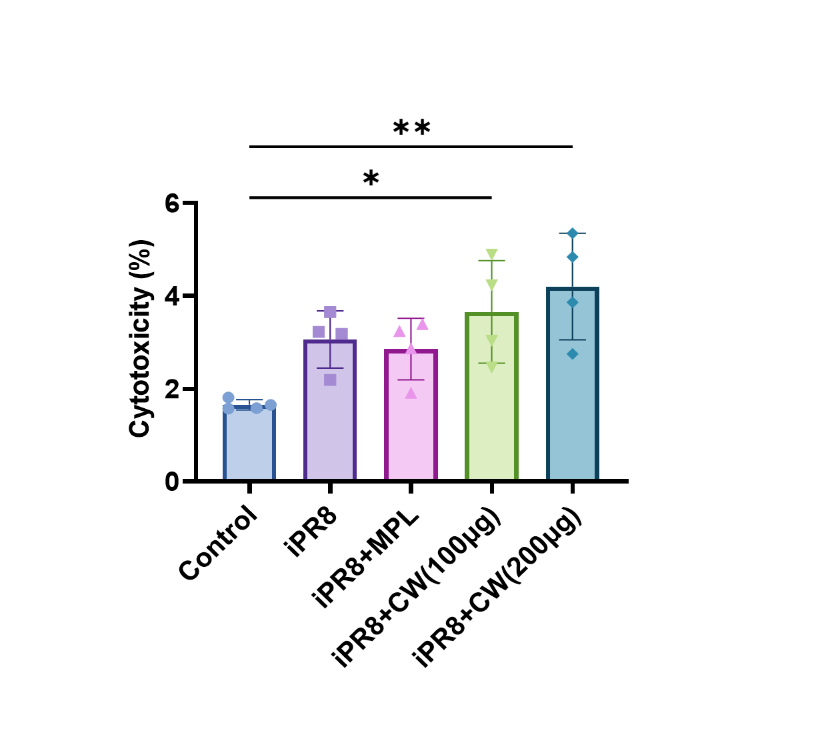
**Supplementary Figure 1. Evaluation of cytotoxicity of CW in NK cells.** NK cells (2x10^5^ cells per well in a 96-well plate) were treated with different concentrations of CW (from 0.095313 to 200 μg/mL) and MPL (0.1 μg/mL). After 2 days of incubation, cell viability was assessed using the Cell Counting Kit-8 (CCK-8, Dojindo, Japan). Briefly, 10 μL of CCK-8 solution was added to each well, followed by a 30 minute incubation at 37°C in 5% CO2. Absorbance was then measured at 450 nm using BioTek Synergy LX Multi-Mode Reader (USA). Data are presented as mean ± SD.

**Supplementary Figure 2. Cytotoxicity mediated by NK cells after in vivo immunizations**. NK cells were isolated from the spleens of mice on day 1 post-boost immunization. YAC-1 target cells were labeled with 2 μM CFSE and cultured with NK effector cells from immunized mice at a 5:1 ratio (E:T). After 4 hours, cells were stained with Live/dead marker, and the frequency of dead target cells (CFSE^+^Live/dead^+^ YAC-1 cells) was assessed by flow cytometry. All data are expressed as mean ± SD. Statistical analysis between groups was performed using one-way ANOVA and Tukey’s multiple comparison test. * p < 0.0332, ** p < 0.0021, *** p < 0.0002, and **** p < 0.0001.


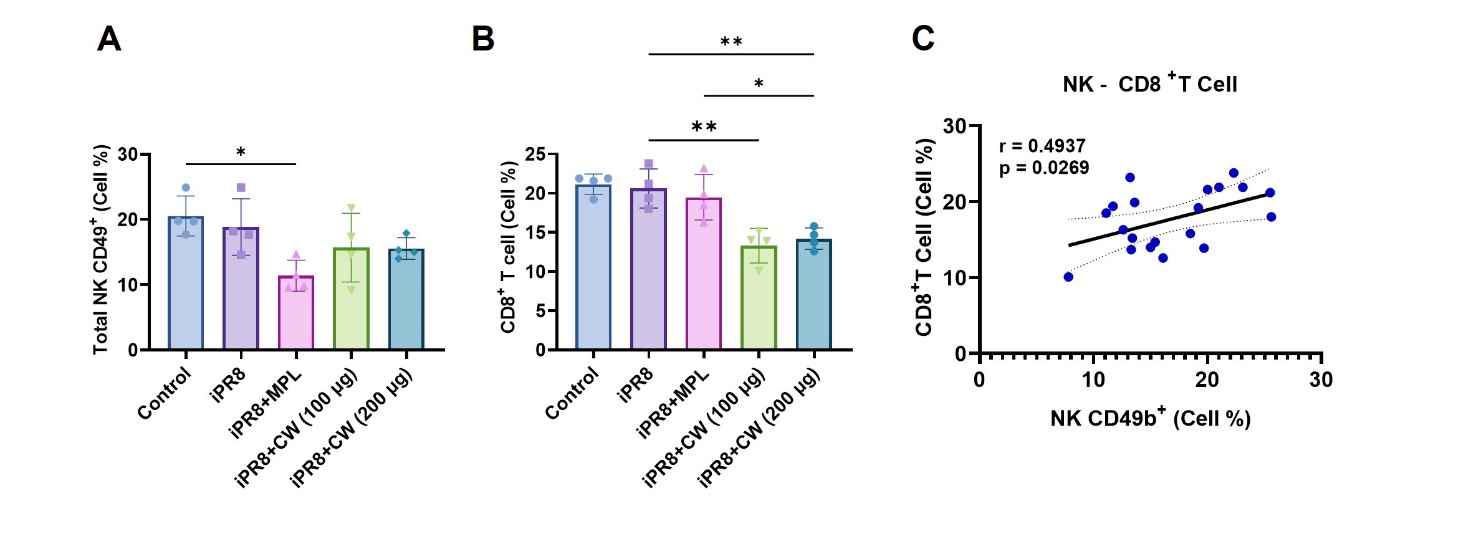


**Supplementary Figure 3**. **Total CD8^+^ T cell population in lung cells after boost vaccination and correlation total NK cell.** Lung samples were collected at 1 day post boost vaccination and the cell percentage in NK cells (A) and CD8^+^ T cells (B) were measured by flow cytometry. The correlation analysis between the CD8^+^ T cell and percentage of NK cell in lung(C). All data are expressed as mean ± SD. Statistical analysis between groups was performed using one-way ANOVA and Tukey’s multiple comparison test. * p < 0.0332, ** p < 0.0021, *** p < 0.0002, and **** p < 0.0001. Pearson’s correlation coefficient was used to analyze the correlations between groups. p < 0.05 was considered statistically significant.

**Supplementary Table 1. List of antibodies used for flow cytometry analysis**

| **Purpose** | **Marker** | **Fluorochrome** | **Clone** | **Supplier** |
| --- | --- | --- | --- | --- |
| DC Activation | LIVE/DEAD™ | Aqua |  | Thermo Fisher Scientific |
|  | CD11c | PE/Cy7 | N418 | BioLegend |
|  | CD40 | BV605 | 3/23 | BD Biosciences |
|  | CD86 | FITC | GL1 | BD Pharmingen™ |
|  | MHC-II | PE | M5/114.15.2 | BioLegend |
| NK Cell Surface Activation | LIVE/DEAD™ | Aqua |  | Thermo Fisher Scientific |
|  | CD49b (DX5) | PE | HMα2 | BD Pharmingen™ |
|  | CD69 | AF700 | H1.2F3 | BD Pharmingen™ |
|  | CD107a | APC | 1D4B | BD Pharmingen™ |
|  | CD3 | BV421 | 17A2 | BioLegend |
|  | CD45 | PerCP | 30-F11 | BioLegend |
| NK Cell Intracellular Staining | IFN-γ | APC/Cy7 | XMG1.2 | BioLegend |
|  | Granzyme B | FITC | GB11 | BioLegend |
| T Cell Intracellular Staining | LIVE/DEAD™ | Aqua |  | Thermo Fisher Scientific |
|  | CD3 | BV421 | 17A2 | BioLegend |
|  | CD45 | PerCP | 30-F11 | BD Pharmingen™ |
|  | CD8a | APC | 53-6.7 | BioLegend |
|  | IFN-γ | APC/Cy7 | XMG1.2 | BioLegend |
|  | Granzyme B | FITC | GB11 | BioLegend |
